# Where the Ockenden report goes wrong: Let us keep calm and follow the evidence

**DOI:** 10.1111/1471-0528.17276

**Published:** 2022-08-22

**Authors:** Ank de Jonge, Raymond De Vries, Eugene Declercq

**Affiliations:** ^1^ Midwifery Science Amsterdam University Medical Centre, Vrije Universiteit Amsterdam Amsterdam The Netherlands; ^2^ Midwifery Academy Amsterdam Groningen, InHolland Amsterdam The Netherlands; ^3^ Amsterdam Public Health, Quality of Care Amsterdam The Netherlands; ^4^ Department of General Practice & Elderly Care Medicine, University Medical Centre Groningen University of Groningen Groningen The Netherlands; ^5^ Center for Bioethics and Social Sciences in Medicine University of Michigan Ann Arbor Michigan USA; ^6^ Midwifery Academy Maastricht Zuyd University Maastricht The Netherlands; ^7^ School for Public Health and Primary Care (CAPHRI) Maastricht University Maastricht The Netherlands; ^8^ Boston University School of Public Health Boston Massachusetts USA

## 1 INTRODUCTION

The Ockenden report is a shocking read.^1^ Families were let down by maternity services resulting in avoidable deaths of women and babies. These tragic events cannot be reversed, but if we are to prevent them from happening again, we must have a clear picture of what went wrong. As researchers from outside the United Kingdom, we were surprised to read some of the recommendations in the report and particularly disappointed by the interpretation of the report in the media. These recommendations and the media reaction may, in fact, aggravate the very problems they are meant to allay.

## 2 SHORTAGE OF STAFF

The report makes quite clear that a shortage of staff is a critical contributor to poor quality of care. Several other problems are, at least in part, a consequence of not having enough people to do the job: junior staff could not be properly supervised, overwork created tension between staff within units and between midwife‐led and obstetric units, a lack of time prevented the provision of personalised care. Junior doctors and midwives were discouraged from seeking assistance and had to wait for help, even in cases where this was urgently needed. The labour ward coordinator is supposed to be available as a back‐up, but was often given a caseload to manage because of the lack of staff, so preventing junior midwives from getting help even when they persistently asked. Midwives were pulled away from midwife‐led units to assist in the obstetric unit, leaving midwife‐led units severely understaffed.

Many of the recommendations in the report make perfect sense, for example, the need to invest in maternity care, employ more staff and reduce attrition of midwives and doctors. Others, like forgoing the monitoring of caesarean section rates, making central cardiotocography (CTG) monitoring systems mandatory and suspending Continuity of Care are more difficult to understand.

## 3 CAESAREAN SECTIONS

One of the recommendations in the report is to stop using total caesarean section percentages as a metric for maternity services. A caesarean section is often a life‐saving procedure, and we would not recommend specifying a ‘target’ rate. However, the World Health Organization continues to advise the assessment, monitoring and comparison of caesarean section rates within healthcare facilities over time.^2^ A caesarean section represents a trade‐off – balancing the risks of major surgery against the medical condition that may place a mother or baby at risk – and it is a decision not to be made lightly. Research has shown that caesarean sections at first births are associated with increased rates of stillbirth in second pregnancies and typically lead to repeat caesarean sections in subsequent births.^3^, ^4^

**FIGURE 1:**
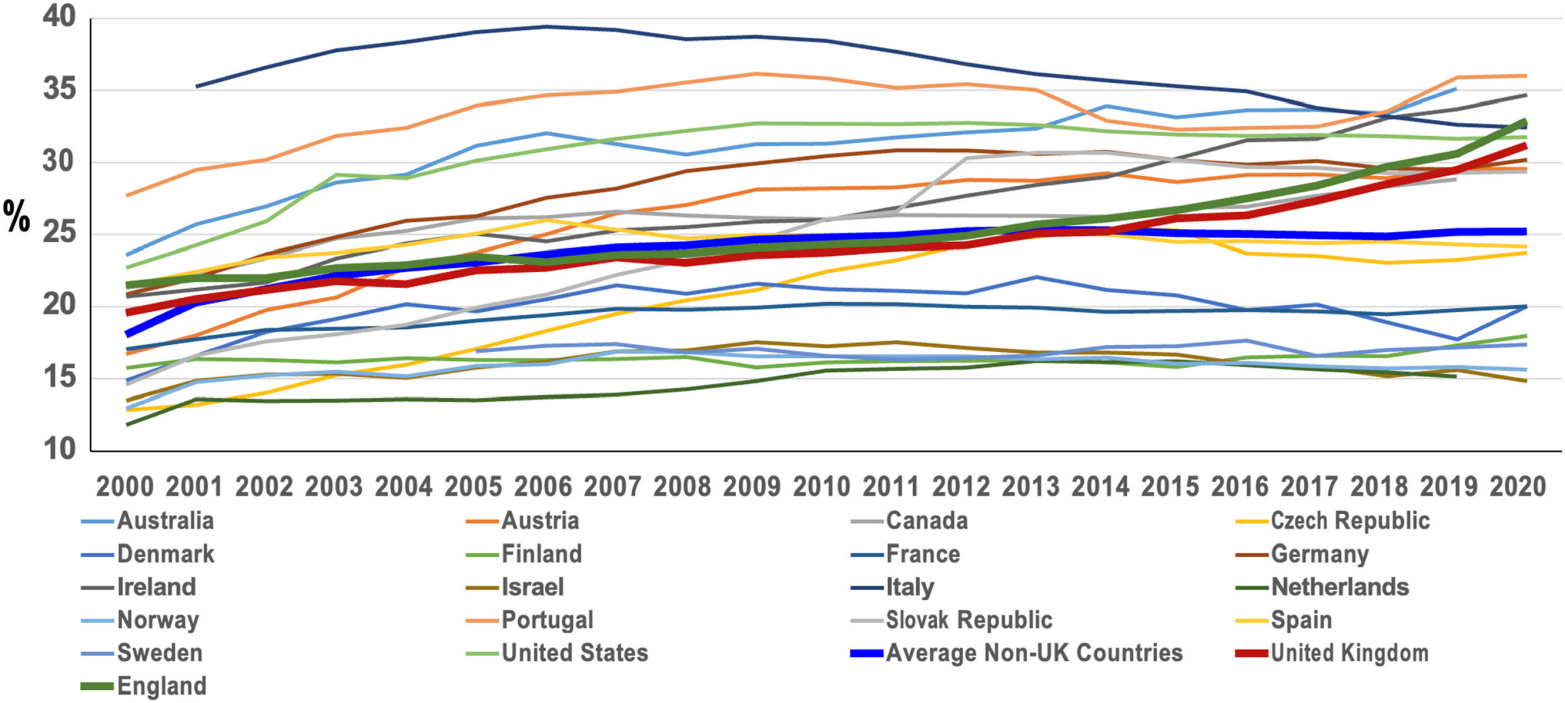
Caesarean trends (%) for Organisation for Economic Co‐operation and Development (OECD) countries, 2000–2020. Source: OECD Health Data 2022; England—NHS Maternity Statistics Annual Reports (2000–2001 through 2020–2021).

In the media, the Ockenden recommendation about caesarean sections has been translated into the assumption that a higher caesarean section rate will lead to lower maternal and perinatal deaths. For example, a headline in the *The Daily Mail* read, ‘Midwives discouraging C‐sections in favour of normal births contributed to many deaths in scandal‐hit trust’ (https://www.dailymail.co.uk/news/article‐10672467/Does‐damning‐Ockenden‐report‐net‐need‐cast‐WIDER.html). Suggesting that efforts to limit caesarean sections were the cause of poor outcomes may have been true in selected individual cases, but it belies the fact that between 2010 and 2020, among wealthy Organisation for Economic Co‐operation and Development (OECD) countries (excluding Mexico and Poland), the United Kingdom experienced the second highest increase in caesarean section rate (31% – from 23.8% in 2010 to 31.2% in 2020), behind only Ireland (33% – from 26.0 in 2010 to 34.7% in 2020). Using National Health Service data for England, we found an even faster 2010–2020 increase in caesarean section rates (35% – from 24.3% in 2010–2011 to 32.9% in 2020–2021) than Ireland. If this trend were to continue, England or Ireland could soon have the highest caesarean section rate in Europe. Notably, this increase came at a time when rates were declining or levelling off among most comparable countries (Figure 1).^5^

**FIGURE 2:**
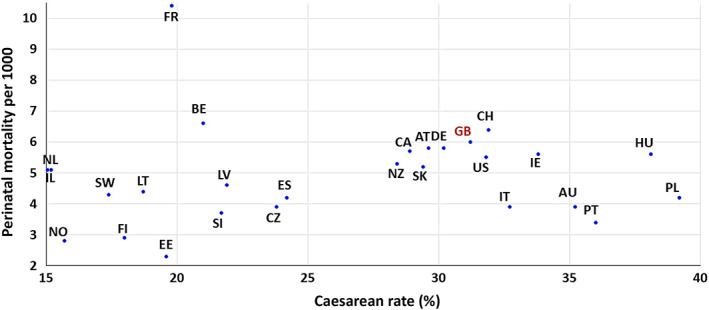
Caesic Co‐operation and Development (OECD) countries, 2019–2020. 2020: AT, Austria; BE, Belgium; CH, Switzerland; CZ, Czech Republic; DE, Germany; EE, Estonia; ES, Spain; FI, Finland; FR, France; GB, United Kingdom; HU, Hungary; IL, Israel; LT, Lithuania; LV, Latvia; NO, Norway; PL, Poland; PT, Portugal; SI, Slovenia; SK, Slovak Republic; SW, Sweden; US, United States. 2019: AU, Australia (provisional value); BE, Belgium; CA, Canada; FR, France; IE, Ireland; IT, Italy; NL, Netherlands; NZ, New Zealand. Source: OECD Health Data 2022.

Is a higher caesarean section rate the key to better outcomes? Figure 2 suggests not. It shows the comparison of caesarean section rates with perinatal mortality rates for 2019 and 2020 in comparable countries. Where 2020 data were not available for a given country, we matched 2019 caesarean sections and perinatal mortality rates. Countries with higher caesarean section rates actually had marginally higher rates of perinatal deaths on average than those countries with lower caesarean section rates. However, the clearest message from Figure 2 is a lack of any guaranteed improvement in perinatal mortality based on a higher caesarean section rate. The tragic case of a poor outcome associated with a delayed caesarean section makes for a powerful media narrative. However, the poor outcomes in the Ockenden Report have more to do with insensitive management and limited staffing than with simple solutions like increasing an already rapidly rising caesarean section rate.

## 4 CONTINUITY OF CARE AND CENTRAL CTG MONITORING SYSTEMS

Surprisingly, one of the recommendations of the report is to review and suspend the Continuity of Care model until all Trusts demonstrate that staffing meets safe minimum requirements on all shifts. There is high‐level evidence of the many benefits of continuity of carer when compared with other models of care, including 24% fewer preterm births and fewer medical interventions.^6^ If organised well, continuity of carer is also associated with lower scores for staff burnout.^7^ Given the fact that 67% of midwives in the United Kingdom suffer from work‐related burnout, investing in continuity of care can help to solve staff shortages.^8^

In a similar vein, the report recommends that centralised CTG monitoring systems be made mandatory in all obstetric units in England. Central monitoring – a technology that allows care providers to see the fetal heart rate tracings and the rate of uterine contractions of all women in labour from one location – seems like a good idea, until you read the research.

We know that the use of continuous CTG increases the likelihood of an unnecessary caesarean with no offsetting benefit for mother and baby.^9^ When the CTG tracing is monitored by staff at a central location, it can undermine clinical safety by disrupting care^10^ and reducing the time that midwives spend with women in labour.^11^

## 5 SUSTAINABLE SOLUTIONS

As others have pointed out, investing in the maternity care workforce is the most important recommendation of the Ockenden report.^1^ Simply increasing the caesarean section rate and abandoning continuity of care initiatives will not improve maternal and newborn care in the United Kingdom. If implemented, these policies will lead to resources being spent on medical interventions and the treatment of adverse outcomes, like preterm birth, that will inevitably follow. Those resources can be better used to employ the staff needed to implement Continuity of Care models properly nationwide.

We agree that lessons need to be learned from the tragic events at the Shrewsbury and Telford Hospital National Health Service Trust. But why throw out the baby of personal care with the bathwater of mismanagement?

## AUTHOR CONTRIBUTIONS

The article was conceived, written, and approved by AdJ, RdV and ED. ED developed the figures.

## CONFLICT OF INTERESTS

None declared. Completed disclosure of interests form available to view online as supporting information.

## ETHICS APPROVAL

All the data used were country level statistics, publicly available from the OECD. As such, ethics approval was not required.

## Supporting information


ICMJE

## Data Availability

Data derived from public domain resources. The data that support the findings of this study are available on the OECD web page (https://www.oecd.org/health/health‐statistics.htm).

## References

[1] Renfrew MJ , Cheyne H , Burnett A , Crozier K , Downe S , Heazell A , et al. Responding to the Ockenden review: safe care for all needs evidence‐based system change – and strengthened midwifery. Midwifery. 2022;112:103391. 10.1016/j.midw.2022.103391 35676100

[2] Betran AP , Torloni MR , Zhang JJ , Gülmezoglu AM . WHO statement on caesarean section rates. BJOG. 2016;123(5):667–70. 26681211 10.1111/1471-0528.13526 PMC5034743

[3] Moraitis AA , Oliver‐Williams C , Wood AM , Fleming M , Pell JP , Smith G . Previous caesarean delivery and the risk of unexplained stillbirth: retrospective cohort study and meta‐analysis. BJOG. 2015;122(11):1467–74. 26033155 10.1111/1471-0528.13461

[4] Zhang JW , Branch W , Hoffman M , De Jonge A , Li SH , Troendle J , et al. In which groups of pregnant women can the caesarean delivery rate likely be reduced safely in the USA? A multicentre cross‐sectional study. BMJ Open. 2018;8(8):e021670. 10.1136/bmjopen-2018-021670 PMC6078266 30082355

[5] Declercq E , Cabral H , Ecker J . The plateauing of cesarean rates in industrialized countries. Am J Obstet Gynecol. 2017;216(3):322–3. 27890649 10.1016/j.ajog.2016.11.1038

[6] Sandall J , Soltani H , Gates S , Shennan A , Devane D . Midwife‐led continuity models versus other models of care for childbearing women. Cochrane Database Syst Rev. 2016;4:CD004667. 27121907 10.1002/14651858.CD004667.pub5 PMC8663203

[7] Newton MS , McLachlan HL , Willis KF , Forster DA . Comparing satisfaction and burnout between caseload and standard care midwives: findings from two cross‐sectional surveys conducted in Victoria, Australia. BMC Pregnancy Childbirth. 2014;14:426. 25539601 10.1186/s12884-014-0426-7 PMC4314764

[8] Hunter B , Fenwick J , Sidebotham M , Henley J . Midwives in the United Kingdom: levels of burnout, depression, anxiety and stress and associated predictors. Midwifery. 2019;79:102526. 31473405 10.1016/j.midw.2019.08.008

[9] Alfirevic Z , Devane D , Gyte GML , Cuthbert A . Continuous cardiotocography (CTG) as a form of electronic fetal monitoring (EFM) for fetal assessment during labour. Cochrane Database Syst Rev. 2017;2:CD006066. 10.1002/14651858.CD006066.pub3 16856111

[10] Small K , Sidebotham M , Gamble J , Fenwick J . “My whole room went into chaos because of that thing in the corner”: unintended consequences of a central fetal monitoring system. Midwifery. 2021;102:103074. 34218022 10.1016/j.midw.2021.103074

[11] Brown J , McIntyre A , Gasparotto R , McGee TM . Birth outcomes, intervention frequency, and the disappearing midwife‐potential hazards of central fetal monitoring: a single center review. Birth. 2016;43(2):100–7. 26865421 10.1111/birt.12222

